# Genome-wide analysis reveals TET-and TDG-mediated 5-methylcytosine oxidation dynamics

**DOI:** 10.1186/1756-8935-6-S1-P88

**Published:** 2013-03-18

**Authors:** Li Shen, Hao Wu, Dinh Diep, Ana C D’Alessio, Alan Fung, Kun Zhang, Yi Zhang

**Affiliations:** 1 Howard Hughes Medical Institute, Harvard Medical School, WAB- 149G, 200 Longwood Ave., Boston, MA 02115, USA; 2 Program in Cellular and Molecular Medicine, Boston Children’s Hospital, Harvard Medical School, WAB- 149G, 200 Longwood Ave., Boston, MA 02115, USA; 3 Department of Genetics, Harvard Medical School, WAB- 149G, 200 Longwood Ave., Boston, MA 02115, USA; 4 Harvard Stem Cell Institute, Harvard Medical School, WAB-149G, 200 Longwood Ave., Boston, MA 02115, USA; 5 Department of Stem Cell and Regenerative Biology, Harvard University, 7 Divinity Street, Cambridge, MA 02138, USA; 6 Departments of Bioengineering, University of California at San Diego, La Jolla, California, USA

## 

Ten-eleven translocation (Tet) family of DNA dioxygenases converts 5-methylcytosine (5mC) into 5-hydroxymethylcytosine (5hmC), 5-formylcytosine (5fC), and 5-carboxylcytosine (5caC) through iterative oxidation reactions. While 5mC and 5hmC are relatively abundant, 5fC and 5caC are at very low levels in the mammalian genome. Thymine DNA glycosylase (TDG) and base excision repair (BER) pathways can actively remove 5fC/5caC to regenerate unmethylated cytosine, but it is unclear to what extent and at which part of the genome such active demethylation processes take place. Here, we have performed high-throughput sequencing analysis of 5mC/5hmC/5fC/5caC-enriched DNA using modification-specific antibodies and generated genome-wide distribution maps of these cytosine modifications in wild-type and *Tdg*-deficient mouse embryonic stem cells (ESCs). We observe that the steady state 5fC and 5caC are preferentially detected at repetitive sequences in wild-type mouse ESCs. Depletion of TDG causes marked accumulation of 5fC and 5caC at a large number of distal gene regulatory elements and transcriptionally repressed/poised gene promoters, suggesting that Tet/TDG-dependent dynamic cycling of 5mC oxidation states may be involved in regulating the function of these regions. Thus, comprehensive mapping of 5mC oxidation and BER pathway activity in the mammalian genome provides a promising approach for better understanding of biological roles of DNA methylation and demethylation dynamics in development and diseases.

